# Evaluation of functional outcome between cemented and uncemented total hip replacement

**DOI:** 10.6026/973206300214121

**Published:** 2025-11-15

**Authors:** Wasim Ahmed, Nishant Kashyap, Rakesh Kumar, Indrajee Kumar, Santosh Kumar, Jawed Akhtar Md

**Affiliations:** 1 Department of Orthopaedics, Indira Gandhi Institute of Medical Sciences, Patna, Bihar, India; 2 Department of Anatomy, Indira Gandhi Institute of Medical Sciences, Patna, Bihar, India

**Keywords:** Functional outcomes, fracture neck of femur, Harris hip score, cemented versus uncemented, total hip replacement

## Abstract

Total hip arthroplasty is an effective procedure for improving mobility and quality of life, but the comparative functional outcomes of cemented and uncemented techniques remain debated. In this prospective randomised study of 50 patients divided into two equal groups, clinical and radiological assessments were performed preoperatively and at regular intervals up to 4 years. Cemented THR demonstrated superior early outcomes, with significantly better pain relief and Harris Hip Scores at 6 weeks and 3 months. By 6 months, functional outcomes between cemented and uncemented THRs converged, with both groups achieving good-to-excellent results and minimal complications. Cemented implants provide faster early recovery, while the long-term advantages of uncemented implants require further investigation to guide individualised implant selection.

## Background:

Total hip replacement (THR) is a widely performed surgical procedure that has provided significant relief to millions suffering from debilitating hip joint pain. Its success lies in its ability to alleviate pain caused by various hip pathologies while preserving joint mobility and stability [1, 2]. The incidence of chronic hip conditions, such as osteoarthritis, inflammatory arthritis and osteonecrosis, is steadily increasing, posing substantial challenges to public health due to associated disability, reduced independence and increased morbidity [3, 4-5]. Since its inception, THR has seen continual advancements in surgical technique and implant technology, leading to improved implant longevity and enhanced functional outcomes. Cemented implants derive their fixation through mechanical interlock between bone and bone cement, whereas uncemented implants achieve primary stability through press-fit fixation and long-term fixation via biological bone ingrowth following initial endosteal microfractures [6]. Both cemented total hip replacement (CTR) and uncemented total hip replacement (UTR) are established surgical techniques; however, the optimal choice between the two remains a matter of clinical debate [6]. Despite significant progress in THR, one fundamental question persists: which method, cemented or uncemented, yields better long-term outcomes? At our institution, as well as many others, uncemented THR is overwhelmingly more common, comprising more than 95% of procedures. In some centres, this figure exceeds 90%. While uncemented prostheses may offer advantages in younger patients due to their longer anticipated lifespan and likelihood of requiring revision, the situation in older patients (particularly those over 50 years) warrants careful consideration. If functional outcomes are comparable, cemented THR, being more cost-effective, may represent a preferable option in this population. To ensure unbiased comparison, this study excluded patients with Dorr type C femora, which are known to influence implant choice and performance. While numerous studies have reported favourable outcomes for both fixation methods, many are constrained by factors such as implant-specific biases, institutional preferences, or limited generalizability due to small sample sizes and short follow-up durations [7, 8-9]. Moreover, population-level data comparing long-term outcomes of CTR and UTR remain scarce [3, 7] and available evidence often suffers from methodological limitations and inadequate follow-up periods [5, 10]. The hip joint, being the primary weight-bearing articulation of the body, plays a critical role in mobility and functional independence. Damage to this joint results in severe impairment of quality of life [11, 12]. THR has emerged as a transformative intervention, offering reliable pain relief and functional restoration [13]. Technological innovations in implant design and fixation have contributed significantly to the durability of modern prostheses. Cemented implants use polymethylmethacrylate (PMMA) to achieve immediate fixation at the bone-implant interface, while uncemented designs aim for long-term stability through biological osseointegration [14, 15, 16-17]. These developments build upon the pioneering contributions of Charnley, whose foundational work in implant biomechanics, materials science and surgical technique laid the groundwork for modern arthroplasty [18, 19, 20-21]. Nonetheless, the debate between cemented and uncemented fixation techniques continues. Younger patients increasingly prefer uncemented implants for their potential longevity and lower revision rates [22, 23]. However, concerns persist regarding their higher initial costs, early revision needs and uncertain long-term durability compared to cemented options [24, 25]. If clinical equivalence in outcomes can be demonstrated, cemented THR may represent a cost-effective and reliable alternative for the older population. In this study, we systematically compare the functional outcomes, assessed using parameters such as the Harris Hip Score and pain scores, between cemented and uncemented THR, excluding Dorr type C femora to ensure valid comparisons. Therefore, it is of interest to provide evidence-based guidance in determining the optimal approach for hip arthroplasty across different patient populations.

## Materials and Methods:

This hospital-based, prospective, randomised, comparative observational study was conducted at the Department of Orthopaedics, Indira Gandhi Institute of Medical Sciences, Patna, Bihar (India) from April 2021 to March 2025. Ethical approval for the study was obtained from the Institutional Ethics Committee of Indira Gandhi Institute of Medical Sciences, Patna (Approval No. 08/IEC/IGIMS/2021). Written informed consent was obtained from all participants prior to their inclusion in the study.

## Sample size:

The sample size was determined using G*Power 3.1 software based on Harris Hip Score (HHS) outcomes from previous studies [26, 27]. With an anticipated 15-point mean difference in HHS (SD=12) between groups, reflecting our pilot data and setting α=0.05 with 80% power for a two-tailed independent t-test, 17 patients per group were required. Accounting for a 20% attrition rate over the 4-year follow-up, we enrolled 25 patients per group (total N=50). This provided adequate power to detect clinically significant differences while accommodating potential dropouts. Thus, a total of 50 patients were enrolled and randomly allocated into two groups (25 patients per group) using the lottery method.

## Exclusion criteria:

Patients were excluded from the study if they had any neurovascular deficits affecting the lower limb, active local or systemic infections such as septic arthritis or osteomyelitis, or if they presented with Dorr type C femora, which are not suitable for cementless fixation due to poor bone quality. Other exclusion factors included severe cardiopulmonary comorbidities that contraindicated surgery, as well as a history of previous ipsilateral hip surgery or multiple fractures.

## Surgical technique: 

In Group 1 (Cemented THR), implants were secured using polymethylmethacrylate (PMMA) bone cement to achieve immediate fixation. In Group 2 (Uncemented THR), press-fit, porous-coated implants were utilised to facilitate biological fixation through bone ingrowth. All surgeries were performed by a single experienced surgeon using the lateral (Modified Hardinge) approach, following standardised surgical protocols to ensure consistency and minimise variability.

## Postoperative follow-up and outcome measures:

Patients were systematically evaluated at multiple intervals following surgery: at 6 weeks to assess wound healing and early mobility; at 3 months for gait analysis and muscle strength evaluation; at 6 months with a focus on radiographic evidence of osseointegration, particularly in the uncemented group; and at 4 years for a comprehensive final functional assessment. The primary outcome measure was functional recovery, assessed using the Harris Hip Score (HHS). Secondary outcome measures included evaluation of pain relief using the Visual Analogue Scale (VAS), documentation of complication rates such as infection, dislocation and aseptic loosening, as well as radiographic assessment to determine implant stability over time.

## Statistical analysis:

All study data were systematically compiled using Microsoft Excel (Office 2019) before being imported into GraphPad Prism version 8.4.3 for comprehensive statistical evaluation. The analytical approach was tailored to variable types: For continuous outcome measures (including Harris Hip Scores and Visual Analogue Scale pain scores), we employed parametric Student's t-tests for normally distributed data. When data violated normality assumptions, we utilised the nonparametric Mann-Whitney U test as a robust alternative. Categorical variables, particularly complication rates and dichotomous outcomes, were analysed using either Pearson's chi-square tests or Fisher's exact tests, with the latter being applied when expected cell frequencies fell below five. Throughout all analyses, we maintained a predetermined threshold for statistical significance at α = 0.05 (two-tailed). This analytical framework ensured appropriate handling of all data types while maintaining rigorous statistical standards for comparative evaluation between the cemented and uncemented THR cohorts.

## Results:

The study population comprised a comparable gender distribution between groups. In Group A (cemented THR), participants included 13 male patients (52%) and 12 female patients (48%). Similarly, Group B (uncemented THR) consisted of 14 male patients (56%) and 11 female patients (44%), demonstrating comparable demographic representation across both cohorts (Figure 1 - see PDF). The comparative analysis of age distribution between the two study groups undergoing total hip replacement (THR) revealed that the mean age in Group A (cemented THR) was 47.96 ± 9.25 years, with an age range of 25 to 65 years. In Group B (uncemented THR), the mean age was slightly higher at 48.97 ± 7.74 years, ranging from 25 to 64.4 years. The overall mean age across both groups was 48.47 ± 8.46 years. A statistical comparison using the p-value showed no significant difference in age distribution between the two groups, with a p-value of 0.677 (Table 1 - see PDF). The cemented THR group comprised 25 patients with varied orthopaedic pathology. Avascular necrosis of the femoral head emerged as the most common indication, affecting nearly half the cohort (48%, 12 cases). Degenerative arthritis accounted for 16% (4 cases), while acute traumatic conditions, including hip fractures, dislocations and femoral neck fractures, each represented 8% of cases (2 patients each). Chronic complications featured non-union of femoral neck fractures in 8% (2 cases) alongside isolated instances of displaced dynamic hip screws (4%). The group also included rare inflammatory conditions, with one case each of rheumatoid arthritis and tuberculous hip involvement. In the uncemented THR group of 25 patients, avascular necrosis demonstrated even greater predominance as the surgical indication, affecting 60% of cases (15 patients). Acute traumatic presentations included femoral neck fractures in 12% (3 cases) and periprosthetic fractures in 4% (1 case). Degenerative arthritis affected 8% (2 patients), while complex revision scenarios encompassed infected bipolar prostheses and intertrochanteric non-unions, both in 4% (1 case) each and femoral neck non-unions occurred in 8% (2 cases) of cases (Figure 2 - see PDF).

The comparison of postoperative pain scores between the two groups showed that patients in the cemented total hip replacement group (Group A) consistently reported more pain relief than those in the uncemented group (Group B) across all follow-up periods. The postoperative pain scores were assessed by using the Visual Analogue Scale (VAS) (scored from 0, indicating no pain, to 10, representing the worst pain imaginable). At 6 weeks, Group A had significantly lower pain than Group B (p = 0.0003). This trend continued at 3 months (p = 0.0003) and 6 months (p = 0.0111). Pain decreased over time in both groups, but cemented THA consistently showed lower scores, suggesting better early-to-mid-term pain control. These findings suggest more effective early stabilisation and pain control with cemented THR (Table 2 - see PDF, Figures 3 - see PDF and 4 - see PDF). The comparison of postoperative function scores between the cemented (Group A) and uncemented (Group B) total hip replacement groups showed that patients in the cemented group had better functional recovery during the early stages of follow-up. At six weeks, Group A had a higher mean function score of 31.79 compared to 26.85 in Group B, a difference that was statistically significant (p = 0.004). This advantage persisted at three months, with Group A showing a mean score of 39.26 versus 32.31 in Group B (p < 0.0001). However, by six months, the difference between the two groups narrowed considerably, with Group A scoring 41.46 and Group B scoring 40.01, and this difference was not statistically significant (p = 0.162) (Table 3 - see PDF). The comparison of Harris Hip Scores between the cemented (Group A) and uncemented (Group B) total hip replacement groups showed that patients in the cemented group experienced better hip function and mobility in the early postoperative period. At six weeks, the mean score in Group A was 76.23, while it was significantly lower in Group B at 61.68 (p = 0.001). This trend continued at three months, with Group A scoring 88.63 compared to 73.22 in Group B (p < 0.0001), indicating a faster recovery in the cemented group. However, by six months, the difference in Harris Hip Scores between the two groups became minimal, 93.19 in Group A and 90.85 in Group B and was not statistically significant (p = 0.356) (Table 4 - see PDF). The Harris Hip Score (HHS) is classified into four categories based on the total score: poor (<70), fair (70-79), good (80-89) and excellent (90-100). In our study, a significant proportion of patients in both groups achieved favourable outcomes. Specifically, 89% of patients in the cemented group achieved scores in the good to excellent range, indicating strong functional recovery. Similarly, 83% of patients in the uncemented group also demonstrated good to excellent outcomes, reflecting a high overall success rate for both surgical techniques.

In the present study, no cases showed any radiological signs of implant loosening or periprosthetic osteolysis during the follow-up period. However, it is well established that complications such as aseptic loosening and osteolysis typically manifest over a longer duration. Therefore, the current follow-up period of four years is relatively short to draw definitive conclusions regarding long-term implant survival and longer-term studies are needed to better assess these outcomes. Intraoperative and postoperative complications observed across both cemented and uncemented total hip replacement groups were minimal. In the uncemented group, one patient experienced excessive intraoperative blood loss, which was managed appropriately without further consequence. In the cemented group, one patient (2%) developed foot drop postoperatively. Notably, sensation remained intact and plantar flexion was preserved, likely caused by improper retractor placement. The condition was managed conservatively, and full recovery was achieved within approximately three months. All surgeries were performed using the lateral (Modified Hardinge) approach. Particular attention was given to meticulous repair of the abductor muscles, which likely contributed to the prevention of limping in either group. These findings suggest that with careful surgical technique and appropriate postoperative care, complications following both cemented and uncemented total hip replacements can be effectively minimised.

## Discussion:

Total Hip Replacement (THR) has proven to be a highly effective surgical solution for patients suffering from severe hip joint damage. Over the years, advancements in implant design and surgical techniques have significantly improved outcomes. However, despite these innovations, the debate over whether cemented or uncemented THR offers superior results remains ongoing. This question is particularly relevant in elderly patients and in developing countries like India, where cost-effectiveness plays a vital role in healthcare decisions. Globally, there has been a shift toward uncemented THR, especially over the past decade. This approach was initially favoured to address certain complications associated with cemented implants, particularly in younger, more active individuals. As a result, many institutions now perform uncemented procedures in over 95% of cases. However, this preference is not without scrutiny [26]. Recent studies suggest that cemented implants may offer better long-term outcomes, especially in older adults. For example, Mishra *et al.* [28] compared the functional outcomes of cemented and uncemented total hip replacements and reported that cemented implants provided better early pain relief, faster recovery, and improved short-term functional scores. In contrast, uncemented implants demonstrated superior long-term stability and fewer cement-related complications. Their study concluded that the choice of implant should be individualised based on patient age, bone quality, and activity level. Similarly, Goyal *et al.* [29] also observed that cemented implants provided better early pain relief, improved functional scores, and faster rehabilitation within the initial months post-surgery. However, long-term results showed comparable outcomes between both groups. They concluded that cemented fixation offers superior short-term clinical benefits and cost-effectiveness, particularly in patients requiring early mobilisation. On the other hand, some studies offer a more balanced view. For instance, a meta-analysis by Morshed *et al.* [30] found no significant difference in survival between cemented and uncemented prostheses. Similarly, Zimmerman *et al.* [31] reported that although uncemented implants were more expensive, there were no statistically significant differences in clinical or functional outcomes between the two groups up to 12 months postoperatively. Further supporting the use of cemented THR, Maggs and Wilson [32] noted that cemented implants have a long track record of excellent outcomes. They pointed out that cemented stems can be positioned precisely according to the patient's anatomy, making them suitable in cases with femoral deformity, osteoporosis, or prior radiotherapy. Additionally, pain relief and early mobilisation are reliably achieved with cemented THR, and if revision surgery is needed, the cement-in-cement technique provides a relatively straightforward solution. Importantly, in the current economic climate, cemented THR remains a highly cost-effective option, which is an important consideration in both public and private healthcare systems. The findings of our study are in close agreement with those of Taanam *et al.* [33], who reported comparable short-term functional outcomes between cemented and uncemented total hip replacements. In their prospective study of 60 patients, both groups achieved excellent postoperative improvement in Harris Hip Scores, with mean scores of 85.6 and 87.1 at six months, respectively, and no statistically significant difference between them. We also observed that there was no statistically significant difference between the cemented and uncemented groups at the two-year follow-up mark. However, patients in the cemented group tended to experience better early pain relief and functional recovery, which aligns with existing literature stating that cemented femoral components provide immediate postoperative stability by facilitating a strong bond between the bone, cement and prosthesis [34, 35].

It is important to note that patients with Dorr type C femurs were excluded from our analysis, as these cases typically require different fixation considerations. Overall, the data suggest that cemented THR offers superior short-term outcomes in terms of pain reduction and early mobility. Moreover, cemented implants are generally less expensive than their uncemented counterparts [36], making them a more economical choice, especially for patients in lower-income settings. Given the financial constraints in developing countries like India and considering the satisfactory short-term clinical outcomes, cemented THR remains a viable and effective option for patients aged 50 to 55 years. That said, we must acknowledge that some of the major complications of THR, such as aseptic loosening and periprosthetic osteolysis, typically appear much later in the postoperative course [37]. Thus, a two-year follow-up period may be insufficient to fully capture long-term differences between the two techniques.

## Limitations of the study: 

While our sample size was statistically adequate for evaluating primary functional outcomes, it may have been insufficient to detect rare complications. Additionally, the relatively short follow-up period limits our ability to assess long-term implant survival and late-onset complications. Future multi-center studies with larger cohorts and extended follow-up durations would provide more definitive conclusions regarding the comparative outcomes of cemented versus uncemented total hip arthroplasty.

## Conclusion:

Our study indicates that cemented total hip replacements (THRs) provide superior early functional outcomes in terms of pain relief and mobility within the first three months postoperatively. However, by six months, the outcomes of cemented and uncemented THRs converge. While uncemented implants are theoretically favoured for their biological fixation and potential long-term advantages, our study's 4-year follow-up is insufficient to validate this. Therefore, the choice between cemented and uncemented THR should be guided by patient-specific factors, including age, bone quality, activity level and economic considerations, with further long-term studies required to determine implant survivorship.
